# Agitation Severity and Psychotropic Prescription in Acute Patients With Delirium Superimposed on Dementia

**DOI:** 10.1111/psyg.70171

**Published:** 2026-04-28

**Authors:** Justine Falciola, François R. Herrmann, Christophe E. Graf, Aline Mendes

**Affiliations:** ^1^ Division of Geriatrics and Rehabilitation University Hospitals of Geneva and University of Geneva Geneva Switzerland; ^2^ Cognitive and Behavioral Geriatric Unit, Division of Geriatrics and Rehabilitation University Hospitals of Geneva and University of Geneva Geneva Switzerland

**Keywords:** agitation, behaviour, delirium superimposed on dementia, Pittsburgh agitation scale, psychotropic drugs

## Abstract

**Background:**

We aimed to compare agitation severity in dementia with versus without delirium (DSD vs. DwD); to identify clinical factors linked to agitation severity; and to examine psychotropic prescribing patterns, including clinical correlates.

**Methods:**

We retrospectively analysed 1709 consecutive patients with dementia admitted to acute geriatric units, comparing those with and without delirium. Delirium and dementia were identified from discharge diagnoses documents. Agitation severity was assessed with the Pittsburgh Agitation Scale (PAS) during the first 72 h. Functional status at admission was documented according to the Functional Independence Measure (FIM). Psychotropic exposure included antipsychotics and benzodiazepines. Multivariable linear regressions examined associations between psychotropics and PAS; logistic regressions identified predictors of psychotropic prescribing.

**Results:**

DSD occurred in 680 patients (39.8%). Compared with dementia alone, DSD was associated with higher PAS scores (5.3 vs. 4.0, *p* < 0.001), poorer functional status (FIM 48.6 vs. 57.8, *p* < 0.001) and more frequent in‐hospital falls (29.9% vs. 17.5%, *p* < 0.001). Psychotropic use was common in both groups but more prevalent in DSD group (73.5% vs. 56.0%, *p* < 0.001), whereas treatment intensity was similar between the two groups. In regression models, psychotropic exposure was associated with higher PAS scores (*β* = 2.48, 95% CI 1.63–3.34), with haloperidol, quetiapine, lorazepam and alprazolam independently associated with greater agitation. Logistic models showed that delirium, in‐hospital falls and hypoxia significantly increased the likelihood of psychotropic prescribing, while Parkinsonism markedly reduced antipsychotic use.

**Conclusion:**

DSD was linked to more severe agitation and psychotropic use. These findings support PAS‐based monitoring and development of DSD‐specific interventions prioritising non‐pharmacological strategies.

## 1 Introduction

Delirium is highly prevalent in hospitalised older adults, especially in those with pre‐existing dementia, where it is referred to as delirium superimposed on dementia (DSD) [1, 2]. More than one‐third of patients with dementia admitted to acute hospitals develop delirium, reflecting the heightened vulnerability of this population [3]. DSD is consistently associated with adverse outcomes, including accelerated functional decline, longer hospital stays, higher rates of institutionalisation and increased mortality [4].

Among the neuropsychiatric and motoric manifestations of DSD, hyperactive states and agitation are especially consequential. Aberrant vocalisations, motor restlessness, aggressiveness and resistance to care complicate treatment and impose a heavy burden on caregivers and staff [5]. Behavioural and psychological symptoms of dementia (BPSD) are already recognised as major drivers of morbidity and healthcare use [6, 7]; their superimposition with delirium potentially amplifies these risks [6, 8]. Yet, despite its clinical importance, the severity and profile of agitation in DSD have rarely been quantified using scales that specifically assess for behavioural symptoms and severity [9].

Pharmacologic management of agitation in delirium remains controversial [10, 11]. Antipsychotics and benzodiazepines are frequently prescribed, but randomised controlled trials in delirium have not demonstrated consistent benefits and both classes carry well‐documented risks, including oversedation, falls, pneumonia, cerebrovascular events and increased risk of mortality, especially in patients with dementia [12, 13, 14]. International guidelines recommend non‐pharmacological measures as first‐line therapy, reserving psychotropics for situations where severe agitation threatens patient or staff safety [11, 13, 15]. In practice, however, psychotropic prescribing remains widespread and little is known about whether prescribing decisions are shaped by measured agitation severity, by patient vulnerability, by acute clinical triggers or by staff burden [16, 17, 18].

Accordingly, this study focused on patients with dementia admitted to acute geriatric wards, with or without superimposed delirium (DSD and DwD). Our first objective was to determine whether DSD is associated with higher agitation scores compared to DwD. Our second objective was to explore clinical factors associated with agitation severity in patients with dementia, with and without superimposed delirium. Our third objective was to investigate psychotropic prescribing practices in patients with dementia, examining whether prescribing is more frequent in those with DSD and to what extent it is driven by intrinsic vulnerability or acute clinical stressors.

## 2 Methods

### 2.1 Design, Setting and Population

We conducted a retrospective cohort study including all patients with dementia admitted to the acute geriatric units (*N* = 1709) of the University Hospitals of Geneva between January 2022 and December 2024. These units admit consecutive older adults referred either from the emergency department (more than 90%) or from other hospital services. Delirium was assessed at admission using the CAM and confirmed by DSM‐5 criteria as part of routine care; for this study, delirium status was retrospectively abstracted from discharge documents. Dementia status was defined by a documented diagnosis recorded at admission or at discharge. DSD was defined as delirium occurring in patients with documented dementia. The study was conducted in accordance with the ethical standards of the institutional research committee and with the 1964 Declaration of Helsinki and its later amendments. The study protocol was reviewed and approved by Geneva's local Ethics Committee, which granted an exemption from written informed consent because of its retrospective observational design (Project Id = 2024‐00010). In accordance with institutional policy, patients and/or their legal representatives are informed at admission that de‐identified clinical data may be used for quality improvement and research, with explicit options to consent or opt out. For studies based on automated extraction of electronic medical records, an institutional opt‐out filter systematically excludes records of individuals who have declined participation, ensuring compliance with their documented decision.

### 2.2 Data Collection

We retrieved data on demographics, comorbidities and acute diagnoses, including in‐hospital falls. Clinical diagnoses were categorised according to International Classification of Diseases (ICD‐10) codes into predefined groups (dementia, neurological, psychiatric, infectious, metabolic, hypoxia‐related, comorbid and others), as detailed in the Supporting Information. Level of consciousness was assessed with the Glasgow Coma Scale (GCS, range 3–15), which evaluates eye, verbal and motor responses, with lower scores indicating reduced consciousness [19]. Pain intensity was measured with the Visual Analogue Scale (VAS, range 0–10), where 0 represents no pain and 10 the worst imaginable pain [20]. Functional status at admission was evaluated using the Functional Independence Measure (FIM, range 18–126), with higher scores reflecting greater independence in activities of daily living [21]. Agitation was measured with the Pittsburgh Agitation Scale (PAS, range 0–16), recorded three times a day during the first 72 h of admission [22]. The PAS evaluates four behavioural domains—aberrant vocalisations, motor agitation, aggressiveness and resistance to care—each scored from 0 (none) to 4 (severe). The maximum score within the first 72 h was retained for analysis.

Psychotropic medication exposure was extracted from the electronic prescribing records, including haloperidol, risperidone, quetiapine, olanzapine, lorazepam, midazolam, oxazepam and alprazolam. For each agent, three exposure variables were constructed: (1) cumulative dose (mg, mean ± SD), (2) number of days of exposure (mean ± SD) and (3) mean daily dose (mg, mean ± SD). These variables captured the total exposure across the hospitalisation and were analysed at the individual molecule levels.

### 2.3 Statistical Analysis

Baseline characteristics were summarised using means with standard deviations (SD) for continuous variables and counts with percentages for categorical variables. Comparisons between patients with dementia with and without delirium (DSD vs. DwD) were performed using independent‐sample *t*‐tests or Wilcoxon rank‐sum tests for continuous variables and *χ* ^2^ tests for categorical variables, as appropriate. These analyses addressed our first objective, to characterise the clinical severity of DSD in terms of agitation, functional status and adverse outcomes such as falls.

For our second objective, the primary outcome of interest was agitation severity, measured by the maximum PAS score within the first 72 h of admission. To identify clinical factors associated with agitation, we constructed multivariable linear regression models. In a first model, psychotropic exposure was included as a binary variable (≥ 1 psychotropic drug vs. none). In a second model, individual psychotropic agents were entered simultaneously to assess molecule‐specific associations. Candidate covariates included demographics (age, sex), intrinsic vulnerabilities (functional independence, psychiatric factors, parkinsonism, comorbidities) and acute stressors (infections, pneumonia, sepsis, acute heart failure, respiratory failure, hypoxia‐related conditions, in‐hospital falls, pain). To prevent overfitting, least absolute shrinkage and selection operator (LASSO) regression with 10‐fold cross‐validation was used for variable selection before entering covariates into the final models. Regression coefficients (*β*), 95% confidence intervals (CI) and *p*‐values are reported.

For our third objective, psychotropic prescribing was examined using multivariable logistic regression models. The main binary outcome was prescription of at least one psychotropic drug during hospitalisation, with additional models fitted for each specific agent (haloperidol, risperidone, quetiapine, olanzapine, lorazepam, midazolam, oxazepam, alprazolam). Predictor variables were selected using LASSO as above and included agitation severity (PAS), intrinsic vulnerabilities and acute stressors. Results are presented as odds ratios (OR) with 95% CIs.

Model performance was assessed using *R* ^2^ (linear models) and pseudo‐*R* ^2^ (logistic models). All tests were two‐sided and a *p*‐value < 0.05 was considered statistically significant. Analyses were conducted using Stata version 18.5 (StataCorp, College Station, TX, USA).

## 3 Results

### 3.1 Patient Characteristics

Among 1709 patients with dementia, 680 (39.8%) presented with delirium. Patients with DSD had lower Glasgow Coma Scale scores (13.7 vs. 14.2, *p* < 0.001) and higher PAS scores (5.3 vs. 4.0, *p* < 0.001). Patients with DSD had lower pain scores (1.6 vs. 2.1, *p* < 0.001), as well as poorer functional status (FIM 48.6 vs. 57.8, *p* < 0.001). Prevalence of in‐hospital falls was higher (29.9% vs. 17.5%, *p* < 0.001) in the DSD group. Infections, including pneumonia, were more frequent in DSD, whereas acute heart failure was less commonly documented among diagnoses.

Psychotropic drug exposure was more prevalent in DSD (73.5% vs. 56.0%, *p* < 0.001), with a higher mean number of psychotropic molecules used per patient (1.4 vs. 0.8, *p* < 0.001). Haloperidol, quetiapine, lorazepam, midazolam and alprazolam were significantly more prescribed in DSD. However, the cumulative dose, number of days of exposure and mean daily dosage of each psychotropic molecule individually did not differ significantly between DSD and DwD, except for quetiapine that was prescribed at a lower daily dose in those with DSD (10.1 mg vs. 21.2 mg, *p* = 0.036). These results suggested that delirium status influenced the likelihood of prescribing but not the intensity of treatment once initiated (Table 1).

**TABLE 1:** Baseline characteristics, agitation severity, psychotropic prescribing and clinical outcomes in patients with dementia with and without superimposed delirium (DSD).

	Dementia without delirium (DwD)	Dementia with delirium (DSD)	Total	*p*
*N*	1029 (60.2%)	680 (39.8%)	1709 (100.0%)	
Female *n* (%)	600 (58.3%)	364 (53.5%)	964 (56.4%)	0.0511
Age y mean (SD)	84.3 (7.0)	84.1 (6.9)	84.3 (7.0)	0.5025
Glasgow mean (SD)	14.2 (1.5)	13.7 (1.7)	14.0 (1.6)	< 0.0010
PAS mean (SD)	271 4.0 (3.9)	350 5.3 (4.3)	621 4.7 (4.2)	< 0.0010
Aberrant vocalisations mean (SD)	0.7 (1.2)	1.0 (1.4)	0.9 (1.3)	0.0058
Motor agitation mean (SD)	1.4 (1.4)	1.7 (1.3)	1.5 (1.4)	0.0065
Aggressiveness mean (SD)	0.8 (1.3)	1.1 (1.5)	1.0 (1.4)	0.0077
Resistance to care mean (SD)	1.2 (1.4)	1.5 (1.5)	1.4 (1.5)	0.0020
VAS for pain mean (SD)	2.1 (2.2)	1.6 (2.2)	1.9 (2.2)	< 0.0010
FIM mean (SD)	57.8 (26.2)	48.6 (24.9)	54.1 (26.1)	< 0.0010
Number of psychotropic drugs mean (SD)	0.8 (0.9)	1.4 (1.2)	1.0 (1.1)	< 0.0010
At least one psychotropic drug *n* (%)	576 (56.0%)	500 (73.5%)	1076 (63.0%)	< 0.0010
Haloperidol *n* (%)	128 (12.4%)	203 (29.9%)	331 (19.4%)	< 0.0010
Cumulative dose mg mean (SD)	4.7 (7.4)	7.1 (16.4)	6.2 (13.7)	0.1224
Days of exposure mean (SD)	7.8 (11.7)	11.1 (21.4)	9.8 (18.3)	0.1047
Mean daily dose (SD)	0.7 (0.4)	0.7 (0.4)	0.7 (0.4)	0.7957
Quetiapine *n* (%)	350 (34.0%)	349 (51.3%)	699 (40.9%)	< 0.0010
Cumulative dose mg mean (SD)	307.6 (519.5)	328.2 (700.2)	317.9 (616.0)	0.6580
Days of exposure mean (SD)	16.9 (19.2)	20.3 (29.5)	18.6 (25.0)	0.0758
Mean daily dose (SD)	16.1 (21.2)	13.5 (10.1)	14.8 (16.7)	0.0393
Risperidone *n* (%)	61 (5.9%)	66 (9.7%)	127 (7.4%)	0.0036
Cumulative dose mg mean (SD)	8.8 (10.9)	12.6 (17.9)	10.8 (15.0)	0.1638
Days of exposure mean (SD)	17.8 (18.6)	24.8 (26.5)	21.4 (23.2)	0.0882
Mean daily dose (SD)	0.5 (0.3)	0.5 (0.3)	0.5 (0.3)	0.8972
Olanzapine *n* (%)	13 (1.3%)	13 (1.9%)	26 (1.5%)	0.2838
Cumulative dose mg mean (SD)	21.8 (24.0)	29.5 (42.4)	25.7 (34.0)	0.5743
Days of exposure mean (SD)	6.9 (7.5)	10.6 (11.7)	8.8 (9.8)	0.3479
Mean daily dose (SD)	3.2 (1.0)	3.1 (2.3)	3.1 (1.8)	0.9222
Lorazepam *n* (%)	151 (14.7%)	168 (24.7%)	319 (18.7%)	< 0.0010
Cumulative dose mg mean (SD)	6.3 (12.6)	6.0 (11.9)	6.1 (12.2)	0.8510
Days of exposure mean (SD)	9.1 (14.9)	9.9 (19.3)	9.5 (17.3)	0.6892
Mean daily dose (SD)	0.6 (0.3)	0.6 (0.4)	0.6 (0.3)	0.6629
Midazolam *n* (%)	23 (2.2%)	40 (5.9%)	63 (3.7%)	< 0.0010
Cumulative dose mg mean (SD)	16.7 (68.3)	3.3 (3.6)	8.2 (41.4)	0.2287
Days of exposure mean (SD)	2.5 (4.6)	2.3 (2.2)	2.4 (3.3)	0.8346
Mean daily dose (SD)	2.0 (2.9)	1.5 (0.8)	1.7 (1.9)	0.3069
Oxazepam *n* (%)	78 (7.6%)	47 (6.9%)	125 (7.3%)	0.6034
Cumulative dose mg mean (SD)	119.0 (164.0)	173.3 (247.2)	139.4 (200.2)	0.1428
Days of exposure mean (SD)	12.0 (16.3)	16.9 (19.8)	13.8 (17.8)	0.1378
Mean daily dose (SD)	9.9 (3.9)	9.0 (3.3)	9.6 (3.7)	0.1923
Alprazolam *n* (%)	14 (1.4%)	19 (2.8%)	33 (1.9%)	0.0350
Cumulative dose mg mean (SD)	4.5 (5.3)	3.9 (5.9)	4.2 (5.6)	0.7801
Days of exposure mean (SD)	13.3 (16.6)	9.7 (12.5)	11.2 (14.2)	0.4872
Mean daily dose (SD)	0.4 (0.1)	0.4 (0.2)	0.4 (0.1)	0.5151
Recent stroke (cerebral infarction/TIA) *n* (%)	5 (0.5%)	10 (1.5%)	15 (0.9%)	0.0327
Parkinson's disease and parkinsonism *n* (%)	94 (9.1%)	67 (9.9%)	161 (9.4%)	0.6190
Neurological factors *n* (%)	163 (15.8%)	99 (14.6%)	262 (15.3%)	0.4716
Psychiatric factors *n* (%)	237 (23.0%)	145 (21.3%)	382 (22.4%)	0.4066
Sepsis *n* (%)	35 (3.4%)	20 (2.9%)	55 (3.2%)	0.5978
Pneumonia *n* (%)	255 (24.8%)	207 (30.4%)	462 (27.0%)	0.0099
Infections *n* (%)	326 (31.7%)	300 (44.1%)	626 (36.6%)	< 0.0010
Factors associated with hypoxia *n* (%)	58 (5.6%)	49 (7.2%)	107 (6.3%)	0.1899
Acute respiratory failure *n* (%)	80 (7.8%)	53 (7.8%)	133 (7.8%)	0.9882
Acute pulmonary embolism *n* (%)	26 (2.5%)	22 (3.2%)	48 (2.8%)	0.3855
Acute heart failure *n* (%)	219 (21.3%)	109 (16.0%)	328 (19.2%)	0.0070
Acute renal failure *n* (%)	224 (21.8%)	161 (23.7%)	385 (22.5%)	0.3555
In‐hospital falls mean (SD) *n* (%)	1029 0.3 (0.8)	680 0.5 (1.1)	1709 0.4 (1.0)	< 0.0010
In‐hospital falls *n* (%)	180 (17.5%)	203 (29.9%)	383 (22.4%)	< 0.0010
Metabolic Factors *n* (%)	552 (53.6%)	371 (54.6%)	923 (54.0%)	0.7104
Recent femoral fracture *n* (%)	5 (0.5%)	5 (0.7%)	10 (0.6%)	0.5082
Comorbidities *n* (%)	691 (67.2%)	435 (64.0%)	1126 (65.9%)	0.1744
Inflammatory/autoimmune *n* (%)	12 (1.2%)	4 (0.6%)	16 (0.9%)	0.2246

Abbreviations: FIM, functional independence measure; PAS, Pittsburgh Agitation Scale; TIA, transitory ischemic accident; VAS for pain, visual analogue scale for pain.

**FIGURE 1:**
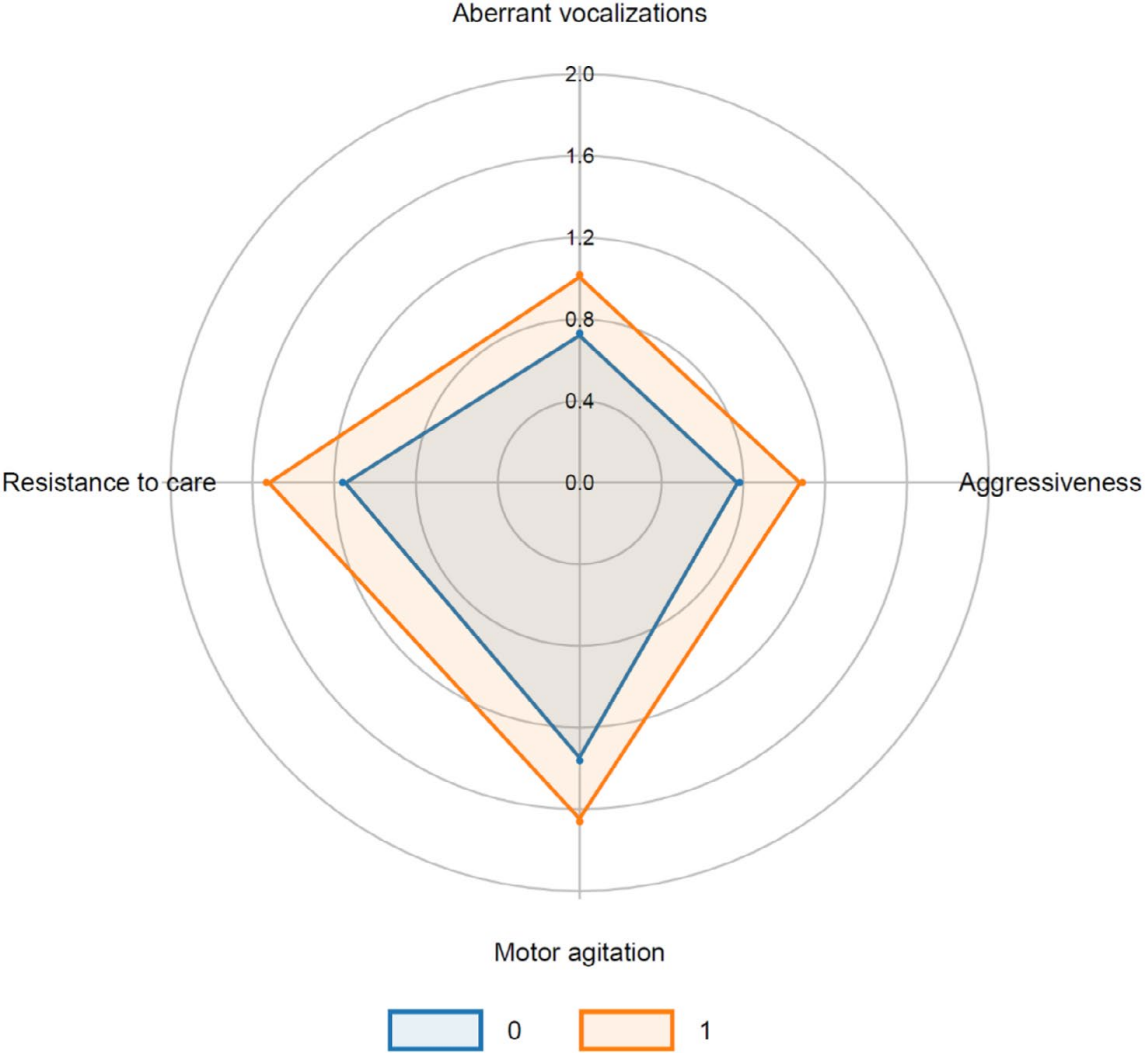
Highest Pittsburgh Agitation Scale (PAS) subscores during the first 72 h of hospitalisation in patients with dementia, comparing DSD (orange) and DwD (blue).

DSD group displayed significantly higher maximum PAS subscores across all behavioural dimensions within the first 72 h of admission, confirming a more severe agitation profile (Figure 1).

### 3.2 Factors Associated With PAS

In multivariable models, having at least one psychotropic prescription was strongly associated with higher PAS scores (*β* = 2.48, 1.63–3.34 95% CI, *p* < 0.001) (Table 2). Specific agents most associated with higher PAS included haloperidol, lorazepam, quetiapine and alprazolam, but not midazolam (Table 3). Clinical correlates with more severe agitation included delirium itself (*β* = 0.85, 0.19–1.50, 95% CI, *p* = 0.011), in‐hospital falls (β = 0.77, 0.78–1.47, 95% CI, *p* = 0.029) and hypoxia‐related conditions (*β* = 1.69, 0.34–3.05, 95% CI, *p* = 0.014). Interestingly, pneumonia was inversely associated with PAS (*β* = −0.94, −1.63 to −0.25, 95% CI, *p* = 0.007). The final multivariable model explained 21% of the variance in PAS scores, indicating modest but clinically relevant explanatory power.

**TABLE 2:** Multivariable linear regression models of factors associated with Pittsburgh Agitation Scale (PAS) scores within the first 72 h of admission, including psychotropic exposure as a binary variable.

PAS^a^	Coeff	95% CI	*p*
At least one psychotropic drug	2.48	1.63–3.34	< 0.001
Delirium	0.85	0.19–1.50	0.011
In‐hospital falls	0.77	0.78–1.47	0.029
Factors associated with hypoxia	1.69	0.34–3.05	0.014
FIM	−0.013	−0.026–0.0004	0.058
Psychiatric factors	0.66	−0.096–1.43	0.087
Pneumonia	−0.52	−1.25–0.21	0.159

Abbreviations: FIM, functional independence measure; PAS, Pittsburgh Agitation Scale.

^a^ *R* ^2^ = 9.5%.

**TABLE 3:** Multivariable linear regression models of factors associated with Pittsburgh Agitation Scale (PAS) scores, including individual psychotropic agents.

PAS^a^	Coeff	95% CI	*p*
Haloperidol	2.92	2.27–3.57	< 0.001
Lorazepam	0.98	0.29–1.67	0.005
Quetiapine	0.98	0.34–1.62	0.003
Pneumonia	−0.94	−1.63–0.25	0.007
Alprazolam	2.11	0.04–4.17	0.045
Factors associated with hypoxia	1.25	−0.02–2.52	0.054
FIM	−0.01	−0.02–0.002	0.098
Psychiatric Factors	0.59	−0.12–1.31	0.103
Delirium	0.51	−0.11–1.12	0.104
Midazolam	0.83	−0.32–1.99	0.158
Risperidone	0.62	0.29–1.67	0.204
In‐hospital falls	0.22	−0.44–0.88	0.516

Abbreviations: FIM, functional independence measure; PAS, Pittsburgh Agitation Scale.

^a^ *R* ^2^ = 21%.

### 3.3 Factors Associated With Psychotropic Prescription

In the overall cohort of patients with dementia, the likelihood of receiving at least one psychotropic medication was nearly doubled among patients with concomitant delirium (OR 1.96, 1.57–2.43, 95% CI, *p* < 0.001) and was also significantly increased in those who presented in‐hospital falls (OR 1.82, 1.39–2.36, 95% CI, *p* < 0.001) and pre‐existing psychiatric diagnoses (OR 1.90, 1.47–2.46 95% CI, *p* < 0.001). By contrast, better functional status at admission, as measured by the FIM, was associated with a lower probability of psychotropic prescription, while the presence of Parkinson's disease or parkinsonism also reduced the odds of receiving such treatment (OR 0.67, 0.48–0.95, 95% CI, *p* = 0.024).

When examining individual agents, distinct patterns emerged (Table 4). Haloperidol use was strongly associated with delirium (OR 2.60, 2.01–3.38, 95% CI, *p* < 0.001), in‐hospital falls (OR 2.66, 2.03–3.50, 95% CI, *p* < 0.001) and sepsis (OR 1.91, 1.03–3.56, 95% CI, *p* = 0.041). However, it was markedly less common in patients with parkinsonism (OR 0.22, 0.11–0.42, 95% CI, *p* < 0.001) or higher comorbidity (OR 0.71, 0.54–0.92, *p* = 0.010). Quetiapine prescriptions were associated with delirium (OR 1.86, 1.51–2.28, 95% CI, *p* < 0.001) and falls (OR 1.87, 1.48–2.37, 95% CI, *p* < 0.001), with a trend towards lower use among patients with higher functional status at admission. Risperidone prescription was associated with psychiatric factors (OR 1.60, 1.05–2.44, 95% CI, *p* = 0.029), but less frequently prescribed among patients with parkinsonism (OR 0.24, 0.09–0.67, 95% CI, *p* = 0.007) and higher functional status (OR 0.99, 0.97–0.99, 95% CI, *p* < 0.001). Lorazepam use was strongly related to in‐hospital falls (OR 1.68, 1.27–2.23, 95% CI, *p* < 0.001) and delirium (OR 1.67, 1.29–2.16, 95% CI, *p* < 0.001), psychiatric factors (OR 1.43, 1.07–1.93, 95% CI, *p* = 0.016), sepsis (OR 1.9, 1.03–3.5, 95% CI, *p* = 0.040) and acute respiratory failure (OR 1.57, 1.02–2.41, 95% CI, *p* = 0.041), but inversely associated with functional status (OR 0.99, 0.98–0.99, *p* < 0.001). Oxazepam was most strongly predicted by psychiatric factors (OR 6.68, 4.47–9.97, 95% CI, *p* < 0.001) and was more often prescribed in men (OR 2.05, 1.38–3.05, 95% CI, *p* < 0.001), while being less frequently given to patients with parkinsonism (OR 0.32, 0.13–0.77, 95% CI, *p* = 0.011) and with older age (OR 0.97, 0.94–0.99, 95% CI, *p* = 0.025). Alprazolam use was also linked to psychiatric factors (OR 3.60, 1.78–7.31, 95% CI, *p* < 0.001) and delirium (OR 2.35, 1.16–4.77, 95% CI, *p* = 0.018). Finally, midazolam prescriptions were also associated with delirium (OR 2.01, 1.17–3.46, 95% CI, *p* = 0.011) and inversely associated functional status (OR 0.97, 0.96–0.99, 95% CI, *p* < 0.001). Pseudo‐*R* ^2^ values indicated modest explanatory capacity, consistent with multifactorial prescribing decisions.

**TABLE 4:** Multivariable logistic regression models of factors associated with psychotropic drug prescribing, overall and by individual agents.

	OR	95% CI	*p*
At least one psychotropic drug, pseudo‐*R* ^2^ = 5.6%			
Delirium	1.96	1.57–2.43	< 0.001
In‐hospital falls	1.82	1.39–2.36	< 0.001
Psychiatric factors	1.90	1.47–2.46	< 0.001
FIM	0.99	0.99–0.99	< 0.001
Parkinson's disease and parkinsonism	0.67	0.48–0.95	0.024
Inflammatory/autoimmune	0.38	0.13–1.08	0.071
Acute pulmonary embolism	1.88	0.94–3.79	0.076
Haloperidol, pseudo‐*R* ^2^ = 11.2%			
In‐hospital falls	2.66	2.03–3.50	< 0.001
Delirium	2.60	2.01–3.38	< 0.001
Parkinson's disease and parkinsonism	0.22	0.11–0.42	< 0.001
FIM	0.99	0.99–0.99	< 0.001
Comorbidities	0.71	0.54–0.92	0.010
Sepsis	1.91	1.03–3.56	0.041
Recent femoral fracture	3.99	0.99–16.14	0.052
Acute pulmonary embolism	1.47	0.74–2.93	0.268
Psychiatric Factors	1.23	0.90–1.67	0.189
Factors associated with hypoxia	1.24	0.76–2.02	0.383
Quetiapine, pseudo‐*R* ^2^ = 4%			
Delirium	1.86	1.51–2.28	< 0.001
In‐hospital falls	1.87	1.48–2.37	< 0.001
FIM	0.99	0.99–1.00	0.056
Age	1.01	0.99–1.03	0.146
Risperidone, pseudo‐*R* ^2^ = 6.5%			
FIM	0.99	0.97–0.99	< 0.001
Parkinson's disease and parkinsonism	0.24	0.09–0.67	0.007
Psychiatric factors	1.60	1.05–2.44	0.029
In‐hospital falls	1.48	0.98–2.24	0.062
Acute renal failure	0.63	0.39–1.04	0.069
Metabolic factors	1.37	0.93–2.01	0.109
Delirium	1.36	0.93–1.99	0.113
Acute pulmonary embolism	1.98	0.85–4.64	0.114
Recent stroke (cerebral infarction/TIA) *n* (%)	2.64	0.69–10.04	0.155
Acute heart failure	0.73	0.43–1.24	0.248
Factors associated with hypoxia	1.50	0.77–2.93	0.237
VAS for pain	0.95	0.86–1.04	0.245
Age	0.99	0.96–1.02	0.480
Lorazepam, pseudo‐*R* ^2^ = 6.2%			
In‐hospital falls	1.68	1.27–2.23	< 0.001
FIM	0.99	0.98–0.99	< 0.001
Delirium	1.67	1.29–2.16	< 0.001
Parkinson's disease and parkinsonism	1.62	1.10–2.40	0.015
Psychiatric Factors	1.43	1.07–1.93	0.016
Sepsis	1.90	1.03–3.50	0.040
Acute respiratory failure	1.57	1.02–2.41	0.041
Acute pulmonary embolism	1.86	0.97–3.57	0.062
Acute heart failure	1.30	0.94–1.80	0.107
Factors associated with hypoxia	1.41	0.88–2.24	0.154
Pneumonia	1.20	0.91–1.60	0.202
Recent femoral fracture	2.30	0.62–8.59	0.215
Metabolic factors	1.16	0.90–1.50	0.261
Inflammatory/autoimmune	0.33	0.04–2.61	0.296
Infections	0.86	0.66–1.13	0.289
Age	0.99	0.98–1.01	0.481
Oxazepam, pseudo‐*R* ^2^ = 17%			
Psychiatric factors	6.68	4.47–9.97	< 0.001
Male sex	2.05	1.38–3.05	< 0.001
FIM	1.01	1.00–1.02	0.043
Age	0.97	0.94–0.99	0.025
Parkinson's disease and parkinsonism	0.32	0.13–0.77	0.011
In‐hospital falls	1.51	0.97–2.35	0.068
Factors associated with hypoxia	0.40	0.14–1.17	0.096
Infections	0.69	0.44–1.08	0.102
Pneumonia	0.69	0.42–1.15	0.152
Recent femoral fracture	2.88	0.50–16.54	0.235
Acute pulmonary embolism	0.27	0.03–2.09	0.208
Comorbidities	0.81	0.53–1.22	0.308
Metabolic factors	1.22	0.82–1.82	0.318
Alprazolam, pseudo‐*R* ^2^ = 9.3%			
Psychiatric factors	3.60	1.78–7.31	< 0.001
Delirium	2.35	1.16–4.77	0.018
VAS for pain	1.13	0.99–1.29	0.055
Acute renal failure	1.93	0.92–4.02	0.080
Male	0.52	0.23–1.13	0.100
Midazolam, pseudo‐*R* ^2^ = 8.8%			
FIM	0.97	0.96–0.99	< 0.001
Delirium	2.01	1.17–3.46	0.011
Acute respiratory failure	2.01	0.98–4.14	0.056
VAS for pain	0.88	0.76–1.02	0.086
In‐hospital falls	1.49	0.86–2.59	0.154
Metabolic factors	1.34	0.79–2.28	0.283

Abbreviations: FIM, functional independence measure; PAS, Pittsburgh Agitation Scale; TIA, transitory ischemic accident; VAS for pain, visual analogue scale for pain.

## 4 Discussion

In this study of 1709 hospitalised patients with dementia, including 680 with DSD, we found that DSD was characterised by more severe agitation, poorer functional status and higher prevalence of in‐hospital falls. Psychotropic drugs were prescribed more frequently in DSD, although treatment intensity (cumulative dose, duration, mean daily dosage of individual psychotropic molecules) was comparable between groups, suggesting that delirium primarily influenced the decision to initiate medication rather than subsequent titration. At the prescribing level, delirium and in‐hospital falls were consistent predictors of psychotropic exposure across drug classes, while reduced functional status at admission increased the likelihood of treatment during hospitalisation. In contrast, parkinsonism markedly reduced antipsychotic use, indicating clinical caution. Importantly, determinants differed by molecule: typical antipsychotics were more often linked to acute infections, while benzodiazepines with psychiatric vulnerability.

Our observational design is vulnerable to confounding by indication: patients with greater behavioural severity or clinical instability are more likely to receive psychotropics and those same features also predict worse outcomes. In our cohort, delirium was established at admission, agitation (PAS) was measured during the first 72 h and psychotropic exposure was defined over the entire hospitalisation. Nevertheless, given that delirium in older adults with dementia often persists for several days, agitation captured within the first 72 h of admission in our study likely reflects behaviour occurring during an ongoing delirium episode, even if precise alignment with delirium severity cannot be confirmed.

Several studies have examined psychomotor subtypes and behavioural manifestations of DSD, though systematic quantification of agitation has been limited. In the Italian Delirium Day study, Morandi and colleagues evaluated 1057 patients with dementia, of whom approximately 370 had superimposed delirium and found that the mixed (34.5%) and hypoactive (33.1%) subtypes predominated, while hyperactive presentations were less frequent (25.6%) but strongly associated with use of restraints and antipsychotics [23]. Schnorr et al. assessed 94 hospitalised patients (43 with DSD, 51 with delirium only) and highlighted circadian and neuropsychiatric disturbances alongside motor features, suggesting that DSD patients express broader behavioural dysregulation than delirium alone [24]. Glynn et al., in a multi‐site cohort of 992 delirium cases, confirmed that those with underlying dementia were less likely to display overt agitation or fluctuation and instead showed more pronounced cognitive and visuospatial deficits [25]. These studies used tools such as the Delirium Motor Subtype Scale [26], the Delirium Rating Scale Revised‐98 [27] and alertness/arousal measures [28], but none specifically deployed agitation‐focused measures. Our application of the PAS, a tool validated for BPSD [22], therefore provides novel granularity in describing the behavioural profile of DSD. The PAS is simple, requires minimal training and is feasible outside specialised units. It can be repeated before and after interventions, enabling clinicians to monitor response in real time. Importantly, PAS scores correlate with the likelihood of severe aggressiveness, reinforcing its clinical relevance for risk stratification in acute geriatric care [29]. In particular, motor agitation and resistance to care were the most prominent PAS domains in patients with DSD. These behaviours are clinically consequential, as they interfere with the timely delivery of diagnostic and therapeutic interventions, potentially delaying correction of delirium precipitants.

Determinants of agitated or hyperactive states in DSD have also been inconsistently explored. Morandi et al. reported associations between hyperactive or mixed subtypes and exposure to urinary or venous catheters, antipsychotic use and antibiotic treatment, while greater functional dependence and severe dementia were linked to non‐hyperactive presentations [23]. Other delirium cohorts, not restricted to dementia, have identified frailty, metabolic disturbances and central nervous system insults as drivers of hyperactive or mixed subtypes [30, 31]. Our study builds on this literature by showing that in‐hospital falls and hypoxia‐related conditions were independently associated with higher PAS scores, even after adjustment for pharmacologic exposure. The association with hypoxia is particularly notable: patients frequently require oxygen therapy, which, although less invasive than urinary catheters, represents a device that may trigger resistance or agitation, in addition to the physiological consequences of hypoxemia itself. Pneumonia, conversely, was inversely associated with PAS, which may indicate that infection‐related delirium in this population more often presents as hypoactive or that delirium was underrecognised in pneumonia cases within our cohort.

Even after adjusting for psychotropic use, falls remained linked to higher PAS scores, suggesting that the relationship is not simply pharmacologically mediated. Mechanistically, agitation may increase risks through motor restlessness, poor safety awareness or impulsivity. Conversely, falls themselves can exacerbate agitation by causing pain, fear of mobilisation or environmental restriction (e.g., bed alarms, closer supervision), which in turn amplify resistance to care. Preventive reflexes may be impaired in DSD, given the coexistence of cognitive impairment, attentional deficits and psychomotor instability, thereby creating a cycle in which agitation and falls reinforce one another. The identification of agitation determinants in DSD also provides essential context for understanding prescribing practices [23].

Evidence on psychotropic prescribing in DSD is limited [12]. Most clinical trials and meta‐analyses have assessed delirium irrespective of dementia status or BPSD [11]. In delirium, randomised controlled trials of antipsychotics have shown no consistent benefit for symptom resolution or duration, while benzodiazepines, outside alcohol or benzodiazepine withdrawal, are discouraged due to risks of oversedation and delirium worsening [32]. Observational studies in dementia cohorts have linked antipsychotics to increased mortality, worse cognitive functioning, cerebrovascular events, pneumonia and falls. Despite these concerns, psychotropic prescribing remains frequent in DSD patients, reflecting both the burden of behavioural symptoms and the paucity of broadly validated effective alternatives.

In our study, psychotropic use was frequent in both groups, affecting more than half of DwD (56%) and nearly three‐quarters of those with DSD (74%). Treatment intensity, however, did not differ substantially, suggesting that delirium acts primarily as a trigger for initiation rather than titration of therapy. This high baseline prevalence underscores the importance of examining how well non‐pharmacological interventions are implemented in practice. Multicomponent bundles, combining orientation, sensory support, mobilisation, hydration and sleep protocols, have been shown in meta‐analyses to reduce incident delirium and falls in hospitalised older adults, although evidence for treating established delirium and specifically DSD is much weaker [33, 34]. Importantly, our study was not designed to assess the appropriateness or guideline concordance of psychotropic prescriptions; rather, it describes real‐world prescribing patterns and their clinical correlates in acute geriatric care.

Psychotropic exposure overall was associated with higher PAS scores and specific agents such as haloperidol, quetiapine, lorazepam and alprazolam were independently correlated with greater agitation. Prescribing determinants varied by molecule: haloperidol and quetiapine were strongly associated with delirium and in‐hospital falls, lorazepam with sepsis and respiratory failure and alprazolam and oxazepam were linked to psychiatric history. The association of benzodiazepines with sepsis and acute respiratory failure likely reflects the urgent need to access care in potentially medically unstable patients, including the use of invasive procedures and monitoring devices that often trigger agitation and resistance. In such situations, clinicians may prefer benzodiazepines for their rapid onset and sedative properties to facilitate urgent interventions. However, this practice carries important risks: benzodiazepines may perpetuate delirium, impair respiratory drive and contribute to adverse outcomes, particularly in frail older adults. On the other hand, Parkinsonism consistently reduced antipsychotic prescribing, reflecting clinical caution. Together, these results suggest that clinicians initiate psychotropics reactively in response to behavioural or safety events and that molecule choice reflects both acute triggers and chronic vulnerabilities.

This study has direct clinical implications. First, agitation severity in delirium superimposed on dementia (DSD), as measured by the Pittsburgh Agitation Scale (PAS), reflects clinically meaningful behavioural dysregulation that is associated with adverse in‐hospital outcomes. PAS therefore provides a practical tool to identify patients at higher clinical risk. Second, psychotropic medications were frequently prescribed and appeared to be initiated primarily in response to agitation severity and safety concerns, rather than through prospective severity‐guided management. In clinical practice, these findings support the use of PAS to monitor the severity and evolution of agitation, to evaluate response to non‐pharmacological interventions and to help identify patients in whom psychotropic treatment may be required when agitation remains severe or poses an immediate safety risk.

To our knowledge, this is the first acute‐hospital study focused on DSD that models molecule‐specific predictors of psychotropic prescribing while concurrently quantifying agitation severity and accounting for acute clinical triggers. This is also the largest cohort of DSD patients studied with systematic behavioural assessment, which strengthens our findings. Although our models explained a modest proportion of variance, this is expected given the multifactorial nature of agitation and prescribing decisions in acute care.

Several limitations warrant consideration. First, the observational design does not allow causal inference and all reported associations should therefore be interpreted as associative. In particular, associations between psychotropic use and agitation severity may reflect confounding by indication and reverse causality, as patients with greater agitation or clinical instability are more likely to receive psychotropic treatment. Delirium was ascertained from discharge documentation, which may have led to underrecognition, especially of hypoactive cases. In addition, the absence of uniform timestamps limits certainty regarding the temporal sequence between agitation peaks and psychotropic prescribing, leaving room for residual and time‐varying confounding. Finally, as a single‐centre study, findings may be influenced by local prescribing practices and may not be fully generalisable.

## 5 Conclusion

In this large, real‐world cohort of hospitalised patients with dementia, DSD was associated with greater agitation within 72 h of admission, poorer functional status, higher in‐hospital falls and a higher likelihood of psychotropic prescribing. Molecule‐specific patterns suggested that prescribing decisions reflect clinical context (delirium, falls, hypoxia, psychiatric history) and contraindication avoidance (Parkinsonism) rather than uniform practice. These findings suggest that psychotropic medications are often initiated reactively in response to agitation or safety events, underscoring the need for systematic agitation monitoring and early non‐pharmacological interventions in patients with DSD.

## Funding

The authors have nothing to report.

## Ethics Statement

The study was conducted in accordance with the ethical standards of the institutional research committee and with the 1964 Declaration of Helsinki and its later amendments. In accordance with Swiss legislation on research involving human subjects, the requirement for informed consent was waived due to the retrospective observational design of the study and the use of routinely collected, de‐identified data. The study protocol was reviewed and approved by Geneva's local Ethics Committee, which granted an exemption from written informed consent because of its retrospective observational design (Project Id = 2024‐00010).

## Conflicts of Interest

The authors declare no conflicts of interest.

## Supporting information


**Data S1:** Supporting Information.

## Data Availability

De‐identified participant data and the study protocol are available upon reasonable request to the corresponding author (aline.mendes@hug.ch) for qualified researchers, subject to institutional and ethical approvals in accordance with Swiss data protection regulations.
