# Supplementary material for: Agitation Severity and Psychotropic Prescription in Acute Patients With Delirium Superimposed on Dementia
Source: Psychogeriatrics. 2026 Apr 28;26:e70171. doi: 10.1111/psyg.70171 (PMC13123300; doi:10.1111/psyg.70171)
Supplement: Supplementary file 1 — Data S1: Supporting Information. [file PSYG-26-0-s001.docx]

**Supplementary Material**

**Categorization of clinical diagnoses according to ICD codes**

**Dementia**

- F01: Vascular dementia
- F02: Dementia in other diseases classified elsewhere
- F02.3: Dementia in Parkinson’s disease
- F02.81: Dementia with behavioral disturbance
- F03: Dementia, unspecified
- G30: Alzheimer’s disease
- G31.83: Dementia with Lewy bodies (DLB)

**Recent stroke (cerebral infarction / TIA)**

- I63.9: Cerebral infarction, unspecified
- G46.8: Other cerebrovascular syndromes
- G45.9: Transient ischemic attack, unspecified

**Parkinson’s disease and parkinsonism**

- G20: Parkinson’s disease
- G21.0: Neuroleptic malignant syndrome
- G21.1: Other drug-induced secondary parkinsonism
- G21.2: Secondary parkinsonism due to other external causes
- G21.3: Postencephalitic parkinsonism
- G21.4: Vascular parkinsonism
- G21.8: Other specified secondary parkinsonism
- G21.9: Secondary parkinsonism, unspecified

**Sepsis**

- A41: Other septicemia
- A41.9: Septicemia, unspecified

**Acute pneumonia**

- J10: Influenza due to identified virus
- J11: Influenza, virus not identified
- J12.8: Other viral pneumonia
- J18: Pneumonia, organism unspecified
- J18.9: Pneumonia, unspecified
- B97.4: Respiratory syncytial virus (RSV) as the cause of diseases classified elsewhere
- U07.1: COVID-19, confirmed by test
- B34.2: Coronavirus infection, unspecified
- J85.0: Gangrene and necrosis of lung
- J85.1: Lung abscess

**Psychiatric factors**

- F10–F19: Mental and behavioral disorders due to substance use
- T50.9: Poisoning by unspecified drug or medicament
- F06.0: Psychotic disorder with hallucinations due to known condition
- F06.2: Psychotic disorder with delusions due to known condition
- F13.4: Sedative/hypnotic withdrawal with delirium
- F32: Depressive episode
- F10.3: Alcohol withdrawal with delirium
- F41: Other anxiety disorders
- F06.4: Organic anxiety disorder
- T43.5: Poisoning by neuroleptics/antipsychotics
- T43.6: Poisoning by other psychotropics
- T42.4: Poisoning by benzodiazepines

**Neurological factors**

- R41.81: Age-related cognitive decline
- R41.89: Other cognitive symptoms / disturbance of consciousness
- G93.4: Encephalopathy, unspecified
- G40: Epilepsy and recurrent seizures
- G31.2: Alcohol-related nervous system degeneration
- F07.1: Post-concussional syndrome
- S06.2: Diffuse brain injury with loss of consciousness
- E53.8: Other vitamin B deficiencies (with cognitive disorders)
- G31.1: Senile cerebral degeneration, NEC
- G23.1: Progressive supranuclear ophthalmoplegia
- G91.9: Hydrocephalus, unspecified
- G93.2: Benign intracranial hypertension
- G91.2: Normal pressure hydrocephalus
- I67.4: Vertebro-basilar insufficiency
- G37.9: Demyelinating disease of CNS, unspecified

**Infections**

- A49.9: Bacterial infection, unspecified
- B95–B96: Bacterial agents causing diseases elsewhere
- L03.0–L03.9: Cellulitis (finger/toe, limb, unspecified)
- R78.81: Bacteremia
- G00, A87, G03.9: Meningitis (bacterial, viral, unspecified)
- A85, G04.9: Viral encephalitis, unspecified
- M46.4–M46.5: Infectious spondylitis/discitis
- M00.9: Pyogenic arthritis, unspecified
- I33.0: Acute and subacute infective endocarditis
- B34.9: Viral infection, unspecified
- K04.6–K12.2: Dental and oral abscesses/cellulitis
- K65.0/K65.9: Peritonitis (acute/unspecified)
- A09: Infectious gastroenteritis/colitis
- B37.0: Oral/esophageal candidiasis
- K75.0: Liver abscess
- N11.0/N10: Pyelonephritis (chronic, acute)
- M86.0–M86.1: Osteomyelitis (acute/chronic)
- M00.0–M00.1: Pyogenic arthritis (staph/other)
- K35.2: Acute appendicitis with generalized peritonitis
- K81.0: Acute cholecystitis
- K83.0: Cholangitis
- N70.0–N71.0: Acute salpingitis/oophoritis; uterine infection
- B37.2: Candidiasis of skin/nails
- B45.0: Pulmonary cryptococcosis
- A18.0: Bone and joint tuberculosis
- A31.0: Pulmonary mycobacterial infection
- A15.0: Confirmed pulmonary tuberculosis
- A50–A64: Sexually transmitted infections (syphilis, etc.)
- B20: HIV with infectious/parasitic diseases
- B25.9: Cytomegalovirus, unspecified
- B39.0: Acute pulmonary histoplasmosis
- N76.4: Vulvar abscess
- G93.0: Brain abscess
- U07.1: COVID-19 confirmed
- N39.0: Urinary tract infection, site unspecified

**Metabolic factors**

- E87.2–E87.8: Acid–base and electrolyte disorders (acidosis, alkalosis, hyponatremia, hyper/hypokalemia, etc.)
- E10–E13: Diabetes with complications (DKA, others)
- E16.0–E16.2: Hypoglycemia/hyperinsulinism
- E03.5: Myxedema coma
- E05.9: Thyrotoxicosis, unspecified
- E06.3–E06.5: Thyroiditis (autoimmune, unspecified)
- E27.2–E27.8: Adrenal insufficiency/crisis, hyperfunction, others
- K72.1: Chronic hepatic failure
- K76.6/K76.9: Portal hypertension; liver disease, unspecified
- N18.6: End-stage renal disease
- E40–E44: Protein-calorie malnutrition (mild to severe)
- E50–E61: Vitamin deficiencies (A, D, niacin, iron, etc.)
- E72.4: Urea cycle disorders
- E88.1: Lipidosis (e.g., Gaucher)
- E22.2: SIADH
- T67.4: Heat stroke

**Recent femur fracture**

- S72.0: Fracture of femoral neck

**Factors associated with hypoxia**

- J80: ARDS
- J96.1/J96.2: Chronic and acute-on-chronic respiratory failure
- J95.2: Postoperative acute pulmonary insufficiency
- J44.0–J45.901: COPD/asthma with acute exacerbation
- I21.9–I25.9: Acute/chronic ischemic heart disease
- G93.1/G93.6: Cerebral anoxia; cerebral edema
- R09.02/R68.81: Hypoxemia; hypoxia, unspecified
- I27.0/I27.2: Pulmonary hypertension (primary/secondary)
- T71.11–T71.19: Mechanical/asphyxia
- Others: hypoxic/circulatory symptoms (R09.89)

**Comorbidities**

- I10: Essential hypertension
- I48.91: Atrial fibrillation, unspecified
- E11.9: Type 2 diabetes, no complication
- E03.9: Hypothyroidism, unspecified
- R54: Frailty/senescence
- C80.1: Primary cancer, site unknown
- Z51.5: Palliative care
- R52: Pain, unspecified
- M62.81: Generalized muscle weakness
- M80–M81: Osteoporosis with/without fracture
- K85.9: Acute pancreatitis, unspecified
- K57.9: Diverticulosis, unspecified
- K74.6: Cirrhosis
- K91.89: Postoperative digestive complications
- M31.0: Takayasu’s arteritis
- N18.5/N18.9: Chronic kidney disease stage 5/unspecified
- M32.9: Systemic lupus erythematosus, unspecified
- C78–C79.3: Metastatic cancer (lung, bone)
- R52.1–R52.2: Chronic pain disorders

**Toxic/iatrogenic**

- T39.9: Poisoning by analgesic/antipyretic
- T44.3: Poisoning by antidepressants
- Z91.19: History of exposure to hazardous agent

**Inflammatory/autoimmune**

- M35.9: Systemic connective tissue disease, unspecified
- D86.9: Sarcoidosis, unspecified
- M35.3: Sjögren’s syndrome

**Environmental and social factors**

- T67.0: Sunstroke
- Z59.1: Food insecurity
- T74.1: Physical abuse

**Other acute conditions**

- Hypoglycemia (E16.0, E16.1)
- Acute hepatic failure (K72.0)
- Acute respiratory failure (J96.0, J96.2)
- Pulmonary embolism (I26.9)
- Acute heart failure (I50.1, I50.9, I50)
- Acute renal failure (N17.9)
